# Ecological Momentary Assessment of Physical Activity and Wellness Behaviors in College Students Throughout a School Year: Longitudinal Naturalistic Study

**DOI:** 10.2196/25375

**Published:** 2022-01-04

**Authors:** Yang Bai, William E Copeland, Ryan Burns, Hilary Nardone, Vinay Devadanam, Jeffrey Rettew, James Hudziak

**Affiliations:** 1 Department of Health and Kinesiology College of Health University of Utah Salt Lake City, UT United States; 2 Vermont Center for Children, Youth, and Families Division of Child Psychiatry University of Vermont Burlington, VT United States

**Keywords:** young adulthood, wellness, substance use, Apple Watch

## Abstract

**Background:**

The Wellness Environment app study is a longitudinal study focused on promoting health in college students.

**Objective:**

The two aims of this study were (1) to assess physical activity (PA) variation across the days of the week and throughout the academic year and (2) to explore the correlates that were associated with PA, concurrently and longitudinally.

**Methods:**

The participants were asked to report their wellness and risk behaviors on a 14-item daily survey through a smartphone app. Each student was provided an Apple Watch to track their real time PA. Data were collected from 805 college students from Sept 2017 to early May 2018. PA patterns across the days of the week and throughout the academic year were summarized. Concurrent associations of daily steps with wellness or risk behavior were tested in the general linear mixed-effects model. The longitudinal, reciprocal association between daily steps and health or risk behaviors were tested with cross-lagged analysis.

**Results:**

Female college students were significantly more active than male ones. The students were significantly more active during the weekday than weekend. Temporal patterns also revealed that the students were less active during Thanksgiving, winter, and spring breaks. Strong concurrent positive correlations were found between higher PA and self-reported happy mood, 8+ hours of sleep, ≥1 fruit and vegetable consumption, ≥4 bottles of water intake, and ≤2 hours of screen time (*P*<.001). Similar longitudinal associations found that the previous day’s wellness behaviors independently predicted the following day’s higher PA except for mood. Conversely, the higher previous-day PA levels were associated with better mood, more fruit and vegetable consumption, and playing less music, but with higher liquor consumption the next day.

**Conclusions:**

This study provides a comprehensive surveillance of longitudinal PA patterns and their independent association with a variety of wellness and risk behaviors in college students.

## Introduction

Research has shown that physical activity (PA) has numerous health and wellness benefits across the life span [1-4]. Despite this, few American college students meet public health recommendations for 150 minutes of moderate and vigorous PA per week [5,6]. Indeed, approximately 40% to 56% of college students participate in PA less than 2 times a week [7]. College students also fail to meet the guidelines for a total of 10,000 steps per day [8,9].

Being able to monitor individuals’ PA plays an important role on PA promotion initiatives [10]. Wearable technologies, such as the Apple Watch (Apple Inc), provide valid estimates of steps, PA time, and energy expenditure under laboratory and free-living conditions, providing opportunities for individuals to self-monitor their behaviors [11-13]. Public health professionals have indicated that wearable devices may be a cost-effective intervention method for PA behavior change to improve health outcomes and facilitate high levels of interest and motivation for PA [14]. However, studies examining the effectiveness of wearable devices in college students are conflicting. Kim et al [15] found that wearing an activity monitor for 15 weeks did not improve PA relative to a control group. However, Pope et al [16] implemented a 12-week, combined smartwatch and health education intervention on a sample of college students, aimed to improve PA, and found statistically significant increases in moderate-to-vigorous physical activity (MVPA) and improvements in other health behaviors or outcomes. The PA level among college students varied dramatically from daily 6-10 minutes to 46-57 minutes, depending on how the PA was measured [15,16].

PA tends to correlate with other health and risk behaviors; therefore, multicomponent approaches to improve health behaviors and lower-risk behaviors may yield greater effectiveness compared with targeting 1 behavior alone [17,18]. The intercorrelation between PA and other health behaviors such as sleep, diet, and water consumption have been extensively explored in both cross-sectional and longitudinal studies where positive associations have been reported [19-21]. Conversely, the associations of risk behaviors such as smoking and alcohol consumption with PA have shown mixed results [22-24].

The longitudinal pattern and correlates of PA in college students may better inform future intervention work for designing ecological and multicomponent health behavior programs [25]. To the best of our knowledge, no study has examined yearlong trajectories of PA using the Apple Watch and correlated the objective PA with other salient health and risk behaviors. Therefore, the primary purpose of this study was to document objective PA trajectories, assessed using the Apple Watch during the 2017-2018 academic year, in a sample of college students. The secondary purpose of this study was to assess the concurrent and longitudinal associations of PA and other health and risk behaviors.

## Methods

### Participants

All participants were from the University of Vermont (UVM) Wellness Environment (WE) study during the 2017-18 academic year. Less than half of the recruited students were assigned to the WE group, and the remainder were assigned to the control group. WE is a neuroscience-inspired health promotion program that incentivizes students to adopt healthy lifestyles. All of the recruited participants had access to a smartphone app, developed to incentivize higher PA, consume a healthy snack after workout, drink more water, and engage in mindfulness activities. WE students were provided resources that included gym access located in the residence halls, group fitness classes, mindfulness classes, and fitness and nutrition mentors.

Study inclusion criteria included full-time UVM undergraduates aged 18-25 years with an iPhone 5 (Appl Inc) or newer (for app compatibility and connection to Apple Watch). A total of 1952 students were originally recruited, and 805 participants (222 Male, 574 Female, and 9 students who chose not to disclose their gender) were included in this study. The study protocol was approved by the UVM Institutional Review Board.

### Instruments and Assessment

#### Apple Watch

All participating students received either a Series 0 or Series 1 Apple Watch. The Apple Watch is equipped with heart rate sensor, accelerometer, and gyroscope to track steps, heart rate, exercise minutes, active and resting energy expenditure, sedentary breaks, distance traveled, and stairs climbed. The students were asked to wear the Apple Watch during the 2017-2018 academic year.

#### Daily Surveys

A 14-item survey was distributed to all participants each night (opening from 7 PM to midnight) via the WE study app on their iPhone or Apple Watch. The survey collected data from 6 wellness behaviors (ie, minutes of exercise, minutes of mindfulness, minutes of music played or sang, fruits and vegetables consumed, hours of sleep, and amount of water consumed) and 7 risk behaviors (ie, cigarette use, consumption of alcoholic drinks, illicit drug use, shots of liquor, number of nonprescribed pills, marijuana use, and hours of screen time). Data informing the overall mood of the day (happy, ok, or sad day) were also included. The daily survey was not a previously validated survey but has been cross-validated with other validated surveys [26]. The participants’ demographic data were also collected at baseline. Apple Watch and daily survey data collected from Oct 9, 2017, to May 13, 2018, were used in the current study, resulting in 216 days of data.

### Data Processing and Analysis

The data were analyzed in 2020. Daily step data were accessed via Apple’s HealthKit application programming interface and screened for compliance. A daily step total of 2000 was used as the wear time cut-off point [27]. The students who had a minimum of 50 valid days of Apple Watch data and completed at least 50% of the daily surveys were included in the final sample. The inclusion criterion of 50 days was the median compliance rate that 50% of the participants (n=1952) had at least 50 days of valid Apple Watch data. Descriptive statistics for demographic variables including age, inclusion in wellness program, gender, race, and year in college as well as average daily steps were computed.

Concurrent associations of daily steps with each wellness or risk behavior were tested first using a general linear mixed effects univariable model (1 behavior as a single predictor). This was followed using a multivariable model (all behaviors entered the same time as multiple predictors) with all 13 wellness and risk behaviors, except for exercise, tested simultaneously.

Longitudinal, reciprocal associations between daily steps and health or risk behaviors were tested with cross-lagged analysis to explore whether the previous-day health and risk behaviors predict the next-day PA and vice versa. The intraclass correlation coefficient was 23.4% in the unconditional model, indicating that 23.4% of the variance in daily steps was between the participants. Thus, all analyses of the daily surveys accounted for repeated, correlated observations within individuals. An autoregressive covariance structure was used, and demographic variables were controlled for all of the mixed models [28]. All analyses were conducted with SAS 9.4 (SAS Institute) software with an alpha value set at *P*<.05.

## Results

### Sample Description

A total of 805 participants with at least 50% daily survey completion and 50 days of valid steps data were included in the analytic sample, which resulted in 77,857 total participant-days’ worth of observations. The analytic sample included in the study did not differ from the baseline sample (N=1871) in terms of demographic distribution (ie, academic year, gender, involvement in the WE program, and race). The average number of observations per participant throughout the 2017-18 academic year was 97 days (SD 48, range 50-212). Overall, the majority of the study sample were female (72.1%), Caucasian (85.4%), and freshman (60.8%) or sophomore (21.8%) (Table 1). No statistical differences were found in the average steps between races, academic class standing, and involvement in the WE program. The only significant difference was that female participants had higher average steps than males (*P*<.001)*.*

**Table 1:** Average daily steps stratified by sample characteristics.

Characteristics and populations			Other values		
			Mean steps	95% CI	*P* value
**Gender, n (%)**					
	Male	222 (27.9)	8488	(8227-8749)	Reference
	Female	574 (72.1)	8904	(8739-9069)	<.001
**WE^a^ status, n (%)**					
	Wellness Environment	351 (43.6)	8833	(8624-9042)	Reference
	College as usual	454 (56.4)	8731	(8545-8917)	.47
**Academic year, n (%)**					
	First year of college	486 (60.8)	8826	(8650-9010)	Reference
	Second year of college	174 (21.8)	8800	(8538-9131)	.98
	Third year of college	113 (14.1)	8539	(8160-8902)	.15
	Fourth year of college	26 (3.3)	8588	(7908-9456)	.71
**Race, n (%)**					
	Caucasian	677 (85.4)	8770	(8624-8927)	Reference
	African American	12 (1.5)	9030	(7992-10,072)	.63
	Asian	53 (6.7)	8714	(8231-9361)	.95
	Latina or Latino	22 (2.8)	8571	(7851-9452)	.77
	Native American	4 (0.5)	8388	(6307-10,469)	.72
	Pacific Islander	3 (0.4)	11,223	(8827-13,620)	.05
	Other	22 (2.8)	8672	(7796-9472)	.74

^a^WE: Wellness Environment.

### Prevalence of Daily Steps

**Figure 1:**
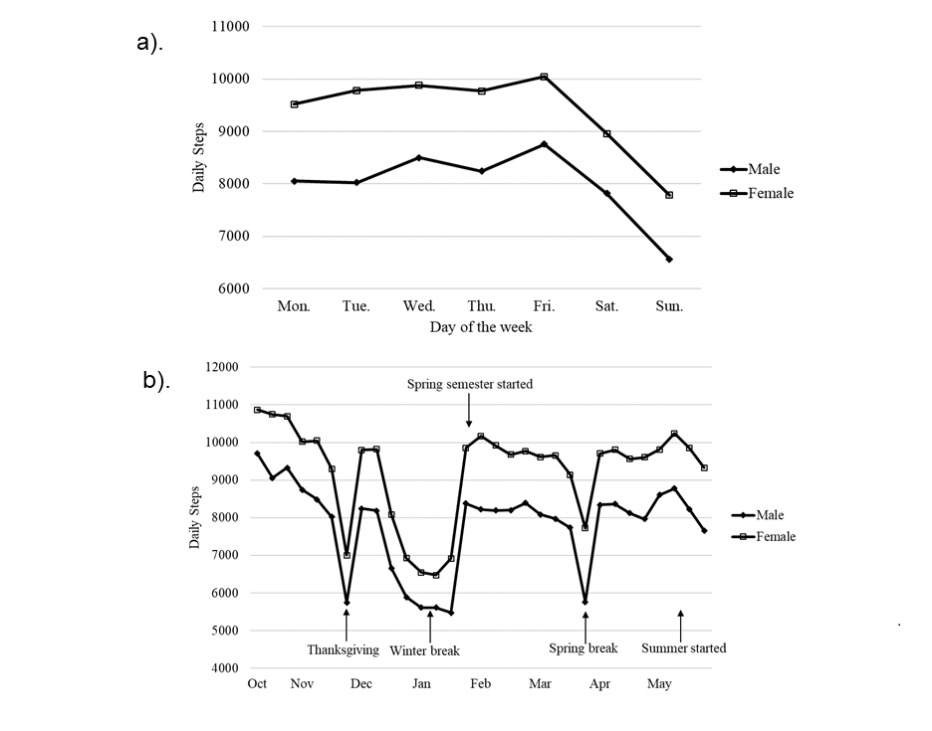
The prevalence of daily steps across day of the week (a) and the academic year (b) by gender.

Steps were tracked daily and across the entire year (Figure 1). The participants were more active during the weekdays (Monday to Friday, ranged from 8989 to 9566), and their daily steps were approximately 1000 fewer on Saturdays (8533, SD 286) and an additional 800 fewer steps on Sundays (7327, SD 286) compared with weekday steps. A larger variation was found across the school year. Step counts were significantly lower during school breaks including Thanksgiving (6999, SD 338), winter break (6471, SD 345), and spring break (7725, SD 341). The most active period was the first 3 weeks of the study, which ranged from 10,697 to 10,864 steps daily. Gender difference attenuated during the weekends and breaks. Compared with males, female participants accumulated about 1500 more steps during weekdays and over 1100 more steps on Saturdays and Sundays.

Note that the daily steps were averaged into weekdays with data from the entire academic year (Figure 1a), and the daily steps were averaged into study weeks (Figure 1b).

### Concurrent Associations of Daily Steps With Wellness or Risk Behaviors

The association of daily steps with 1 wellness or risk behavior (Multimedia Appendix 1: Supplemental Table 1) in the univariable model was similar to that of the steps with multiple behaviors in the multivariable model (Table 2). Higher levels of PA were associated with happy mood (>650 daily steps) and other health behaviors, including ≥1 servings of fruits and vegetables, ≥4 glasses of water, and ≥1 minute of mindfulness practice (*P*<.001). A dose-response association was also found between PA with fruit and water consumption. Nonacademic screen time was negatively associated with daily steps in that a difference of over 2000 steps was found between the participants who spent 0-2 hours and 7+ hours on screen (*P*<.001).

Moreover, 3 out of 6 substance abuse behaviors (ie, liquor, cannabis, and nonprescribed pills) were significantly associated with PA (*P*<.001). For 30 minutes of self-report exercise, there was approximately 1700 additional steps estimated by Apple Watch (*P*<.001). The cumulative wellness items had a positive association with daily steps (*P*<.001). The participants were most active when they had 0 risk behaviors (9338 steps; 95% CI 8748-9928) followed by those who engaged 2 and more risk behaviors (8855 steps; 95% CI 8257-9452) and then 1 risk behavior (8508 steps; 95% CI 7918-9099).

**Table 2:** Healthy and risky behaviors predicting daily steps within the multivariate model.

Healthy and risky behaviors^a^			Estimated steps		95% CI		*P* value
**Mood**							
	Sad	8535		(7774-9295)		Reference	
	Ok	8622		(7867-9378)		.17	
	Happy	9189		(8434-9944)		<.001	
**Sleep (hours)**							
	<4	9194		(8419-9969)		.51	
	4-7	9125		(8372-9879)		Reference	
	8+	8027		(7273-8780)		<.001	
**Fruit, n**							
	0	8169		(7412-8927)		Reference	
	1-3	8798		(8044-9552)		<.001	
	4+	9378		(8619-10,138)		<.001	
**Water, n**							
	0-3	8105		(7349-8861)		Reference	
	4-6	8824		(8069-9579)		<.001	
	7+	9416		(8655-10,177)		<.001	
**Screen time (hours)**							
	0-2	9848		(9094-10,601)		Reference	
	3-6	8859		(8104-9613)		<.001	
	7+	7639		(6871-8408)		<.001	
**Mindfulness (minutes)**							
	0	8386		(7632-9141)		Reference	
	1-9	9009		(8251-9767)		<.001	
	10+	8951		(8191-9711)		.02	
**Music (minutes)**							
	0	8752		(7996-9508)		Reference	
	1-30	8731		(7974-9487)		.64	
	31+	8863		(8106-9620)		.04	
**Alcohol**							
	No	8811		(8057-9565)		Reference	
	Yes	8753		(7992-9513)		.41	
**Liquor**							
	No	8487		(7733-9242)		Reference	
	Yes	9077		(8313-9840)		<.001	
**Marijuana**							
	No	8674		(7919-9430)		Reference	
	Yes	8890		(8128-9651)		.01	
**Cigarettes**							
	No	8724		(7973-9474)		Reference	
	Yes	8840		(8042-9638)		.52	
**Illicit drugs**							
	No	8881		(8219-9544)		Reference	
	Yes	8682		(7660-9704)		.64	
**Nonprescribed pills**							
	No	9169		(8434-9904)		Reference	
	Yes	8395		(7542-9248)		<.001	
**Gender**							
	Male	8519		(7729-9308)		Reference	
	Female	9045		(8287-9803)		<.001	
**WE^b^ status**							
	Wellness Environment	8712		(7918-9506)		Reference	
	College as usual	8852		(8097-9607)		.44	
**Academic year**							
	First year of college	9074		(8333-9816)		Reference	
	Second year of college	8752		(7967-9537)		.12	
	Third year of college	8644		(7819-9468)		.09	
	Fourth year of college	8658		(75552-9764)		.36	
**Race**							
	Caucasian	8360		(7797-8922)		Reference	
	African American	8596		(7264-9927)		.71	
	Asian	8429		(7626-9232)		.83	
	Latina or Latino	8435		(7383-9487)		.87	
	Native American	8110		(5914-10,305)		.82	
	Pacific Islander	11,039		(8502-13,575)		.03	
	Other	8506		(7391-9622)		.77	

^a^Demographic factors were controlled in the model.

^b^WE: Wellness Environment.

### Longitudinal Associations of Daily Steps With Wellness of Risk Behaviors

The previous-day behaviors predicting the next day’s PA were tested using both univariable (Multimedia Appendix 1: Supplemental Table 2) and multivariable models (Table 3). The results indicated that each of the self-reported behaviors independently predicted the next day’s PA. As shown in Table 3, the previous-day fruit and vegetable consumption, water consumption, and mindfulness practice had significantly positive associations with the following day’s PA. The students who had 1-3 and 4+ servings of fruit and vegetables accumulated 656 and 375 more following-day steps compared with students who had no fruit and vegetable consumption, respectively (*P*<.001). Similarly, the students who had 7+ bottles of water accumulated 335 and 215 additional following-day steps compared with those who had 0-3 and 4-6 bottles of water (*P*<.001). The previous-day screen time was a negative predictor of the following-day PA. The students who had 3-6 hours and 7+ hours of screen time accumulated 203 and 506 fewer following-day steps compared with those who had 0-2 hours of screen time (*P*<.001). The students with any mindfulness practice had 300-400 additional following-day steps compared with those who had no mindfulness practice (*P*<.001). However, Students who were happy or played music predicted lower following-day PA compared with those who had a sad mood or played no music. Previous-day cigarettes and illicit drugs did not significantly predict the following day’s PA (Table 3). Conversely, higher previous-day PA levels were associated with less following-day exercise, higher fruit and vegetable consumption, less playing music, and higher liquor consumption (*P*<.05, Table 4).

**Table 3:** Previous-day healthy and risky behaviors predicting daily steps in multivariate model.

Previous-day healthy and risky behaviors^a^			Estimated steps		95% CI		*P* values
Steps			0.2		(0.19-0.21)		<.001
**Mood**							
	Sad	8788		(8033-9543)		Reference	
	Ok	8812		(8064-9559)		.75	
	Happy	8487		(7740-9234)		<.001	
**Sleep (hours)**							
	<4	8600		(7827-9373)		.27	
	4-7	8736		(7991-9481)		Reference	
	8+	8751		(8005-9497)		.70	
**Fruit, n**							
	0	8383		(7633-9134)		Reference	
	1-3	8664		(7918-9411)		<.001	
	4+	9039		(8286-9792)		<.001	
**Water, n**							
	0-3	8544		(7796-9292)		Reference	
	4-6	8664		(7916-9411)		.03	
	7+	8879		(8124-9634)		<.001	
**Screen time (hours)**							
	0-2	8932		(8187-9677)		Reference	
	3-6	8729		(7983-9476)		<.001	
	7h+	8426		(7661-9191)		<.001	
**Mindfulness (minutes)**							
	0	8460		(8140-9642)		Reference	
	1-9	8891		(9066-10,036)		<.001	
	10+	8737		(7983-9490)		<.001	
**Music (minutes)**							
	0	8803		(8055-9551)		Reference	
	1-30	8697		(7947-9446)		.04	
	31+	8587		(7837-9336)		<.001	
**Alcohol**							
	No	8945		(8200-9691)		Reference	
	Yes	8446		(7690-9202)		<.001	
**Liquor**							
	No	8921		(8175-9668)		Reference	
	Yes	8470		(7710-9230)		<.001	
**Marijuana**							
	No	8804		(8055-9553)		Reference	
	Yes	8587		(7832-9342)		.03	
**Cigarettes**							
	No	8612		(7869-9355)		Reference	
	Yes	8779		(7973-9586)		.44	
**Illicit drugs**							
	No	8678		(8071-9286)		Reference	
	Yes	8713		(7587-9839)		.95	
**Nonprescribed pills**							
	No	8932		(8207-9657)		Reference	
	Yes	8459		(7577-9340)		.13	
**Gender**							
	Male	8535		(7766-9304)		Reference	
	Female	8857		(8107-9606)		.03	
**WE^b^ status**							
	Wellness Environment	8595		(7821-9370)		Reference	
	College as usual	8796		(8050-9542)		.18	
**Academic year**							
	First year of college	8852		(8114-9590)		Reference	
	Second year of college	8664		(7897-9431)		.27	
	Third year of college	8444		(7650-9239)		.05	
	Fourth year of college	8822		(7824-9820)		.94	
**Race**							
	Caucasian	8482		(7858-9105)		Reference	
	African American	8472		(7290-9654)		.99	
	Asian	8494		(7715-9274)		.96	
	Latina or Latino	8256		(7290-9221)		.56	
	Native American	7969		(6139-9799)		.56	
	Pacific Islander	10,529		(8414-12,644)		.05	
	Other	8667		(7657-9678)		.65	

^a^Demographic factors were controlled in the model.

^b^WE: Wellness Environment.

**Table 4:** Previous day daily steps predicting healthy and risky behaviors within the univariable model.

	Previous day steps as predictor^a^		
Outcomes	Coefficient	95% CI	*P* value
Mood	-0.0049	(-0.013, 0.0031)	.07
Sleep	-0.0001	(-0.0079, 0.0077)	.98
Exercise	-0.010	(-0.018, -0.0027)	.01
Fruit	0.011	(0.0033, 0.018)	.01
Water	0.0047	(-0.0032, 0.013)	.24
Screen time	0.0070	(-0.001, 0.015)	.09
Mindfulness	-0.0080	(-0.018, 0.0016)	.10
Music	-0.0096	(-0.018, -0.0014)	.02
Alcohol	0.013	(-0.0003, 0.027)	.06
Liquor	0.027	(0.015, 0.038)	<.001
Marijuana	0.0095	(-0.007, 0.026)	.25
Cigarettes	0.011	(-0.013, 0.035)	.36
Illicit drugs	0.029	(-0.006, 0.063)	.11
Nonprescribed pills	-0.011	(-0.038, 0.017)	.45

^a^Demographic factors were controlled in the models.

## Discussion

### Principal Findings

This paper provided a unique and comprehensive profile of objectively measured daily PA and self-reported health and risk behaviors using ecological momentary assessment over 7 months within 1 academic year among college students. We found PA variations between the weekday and weekend and between school days and academic breaks. Gender differences in PA were attenuated during the weekend and academic breaks. Compared with risk behaviors, stronger and independent associations were found between PA and several wellness behaviors including self-report exercise minutes, fruit and vegetable consumption, sleep, water consumption, and mood states.

Although consumer monitors provide the potential to track PA in real time during longer time periods, few studies have reported continuously monitored PA levels (ie, for more than a few weeks) in adult or youth populations. Several studies have applied consumer monitors such as Fitbit (Fitbit Inc) or Misfit (Misfit Inc) in intervention studies lasting from 12 weeks to 6 months, but the majority of the studies have used research-based activity monitors (eg, Actigraph) and only reported baseline and posttest MVPA [15,29]. Three intervention studies used Fitbit and reported the real-time steps or MVPA data over 1 year [30], 12 weeks [31], and 8 weeks [32]. Although direct comparisons to this study are precluded due to the sample and setting differences, there may have been possible behavioral reactivity within in the first 2 or 3 weeks of wearing the device [30-32]. No other prior ecological momentary assessment of PA studies in college students were identified.

Similar to 2 other recent intervention studies with college students using wearables [15,16], around 70% of the participants in this study were female. Mixed results were found in the literature about gender differences and PA levels among college students. The majority of previous research using self-report data suggested that males were more active than females; however, studies using objectively measured PA found either no gender differences or females being more active than males [33-35]. Our study showed that female participants were more active than their male counterparts during the week and across the school year. Possible explanations for these differences could be that males tend to overestimate their PA levels in the self-report data, and they tend to engage in resistance training, which is a nonambulatory activity that may not be captured by the Apple Watch [11,36].

Our study also found that the participants were more active during structured days (ie, school days) than unstructured days (ie, academic breaks and weekends). A few other studies also observed that college students were more active during weekdays than weekends [33,34]. For example, Clemente et al [37] found that both male and female Portuguese college students were more active during weekdays than weekend days. However, this finding was expected because most students had to walk to or around campus during weekdays [34]. Despite this, we did not find empirical studies that examined the seasonal patterns of objective PA in a college sample, which may be due to prior measurement constraints such as the lack of technology to continuously monitor PA over several months with research-based monitors.

Besides environmental changes, university students, especially first-year students, undergo social, academic, emotional, and physiological changes that may influence their lifestyle and behaviors such as higher stress levels leading to increased drug and alcohol abuse [38]. Young adults had the highest prevalence (13.1%) of major depressive episodes compared with other adult age groups, according to the data from 2017 National Survey on Drug Use and Health [39]. Several randomized controlled trials indicated the small-to-moderate therapeutic effects of exercise on depression and anxiety disorders [40,41]. Our study indicated that students who had higher PA had better current and next day mood. Encouraging college students to engage in PA or exercise could be an effective way to cope with academic and interpersonal stress during the transition from high school to college.

The magnitude of association of PA and other wellness and risk behaviors remained generally consistent in the univariable and multivariable models, supporting the independent association between PA and wellness and risk behaviors in college students. For PA and health behaviors, fruit, vegetable, and water consumption were positively correlated with PA levels while screen time was negatively associated with PA, which have been confirmed by other cross-sectional studies [20,21,42]. However, the significant temporal link between PA and fruit or vegetable consumptions found in our study contradicted the findings of previous studies in the literature [19]. Congruent with prior research, our study also found that PA levels decreased from freshman to senior year. This indicates the importance of offering health behavior education and promotion programming to freshmen, targeting several health and risk behaviors [42-44]. Unlike other studies [20,35,42], our study did not find significant associations between PA and risk behaviors. Notwithstanding, PA was identified as a protective factor for alcohol and substance use behaviors in our temporal analysis. A possible explanation is that the majority of the study sample were freshmen who live on campus, half of whom reside in WE housing where students sign a contract not to consume alcohol in their dorm. Thus, those students who wanted to consume alcohol needed to walk to bars, parties, and stores, increasing their daily step count.

### Strengths and Limitations

To the best of our knowledge, this is the first study using an ecological momentary approach to assess multiple wellness behaviors in 1 academic year among college students. The study assessed real time and objective PA within a real-world setting over a full academic year in addition to the tracking of other wellness and risk behaviors. Academic breaks and weekends were identified as inactive periods for college students, which allows targeted interventions to be designed to enhance the health of young adults during this critical life transition period. The successful deployment of the study app allowed us to collect 13 different wellness behaviors and mood states simultaneously on a daily basis. This enabled us to study the different wellness behaviors’ independent and dependent associations as well as concurrent and longitudinal associations with PA.

There are a number of limitations in this study. First, the study sample was generally homogeneous with a majority of participants being female and Caucasian at a single university in Northeastern United States. Second, the Apple Watch provided objectively measured step count data, but no wear time data were available to assess. Considering the possible linear association of longer wear time and higher number of steps accumulated, the activity level could be skewed for students who wore the Apple Watch longer and slept less. Third, the wellness and risk behaviors were measured by self-report daily survey items with fixed response sets.

### Implications and Contribution

The independent associations explored between PA and a variety of other health-related behaviors indicated that promoting one behavior will not necessarily influence other behaviors. This study provided novel information on specific patterns of college students’ objective PA, wellness, and risk behaviors over an academic year.

## Appendix

Multimedia Appendix 1 Healthy and risky behaviors predicting daily steps within the univariable model. Previous day healthy and risky behaviors predicting daily steps within the univariable model.

## Abbreviations

MVPA: moderate-to-vigorous physical activity
PA: physical activity
UVM: University of Vermont
WE: Wellness Environment
