# Supplementary material for: Ecological Momentary Assessment of Physical Activity and Wellness Behaviors in College Students Throughout a School Year: Longitudinal Naturalistic Study
Source: JMIR Public Health Surveill. 2022 Jan 4;8(1):e25375. doi: 10.2196/25375 (PMC8767478; doi:10.2196/25375)
Supplement: Multimedia Appendix 1 [file publichealth_v8i1e25375_app1.docx]

Supplemental Table 1. Healthy and risky behaviors predicting daily steps within the univariable model.

|  |  | N (%) | Estimate | 95% CI | *p* |
| --- | --- | --- | --- | --- | --- |
| Mood | Sad | 5392 (6.9%) | 8586 | (7988, 9184) | Ref |
|  | Ok | 32570 (41.8%) | 8683 | (8094, 9272) | 0.13 |
|  | Happy | 39884 (51.2%) | 9282 | (8693, 9871) | <.0001** |
|  |  |  |  |  |  |
| Exercise | No exercise | 18187 (23.4%) | 6969 | (6394, 7544) | Ref |
|  | 1-30mins daily | 20134 (25.9%) | 7933 | (7358, 8507) | <.0001** |
|  | 31-60 mins daily | 21483 (27.6%) | 9629 | (9055, 10203) | <.0001** |
|  | 60+ mins daily | 17967 (23.1%) | 11306 | (10731, 11880) | <.0001** |
|  |  |  |  |  |  |
| Sleep | <4h | 1775 (2.3%) | 9359 | (8740, 9978) | 0.38 |
|  | 4-7 h | 41179 (52.9%) | 9446 | (8855, 10037) | Ref |
|  | 8+ h | 34861 (44.8%) | 8343 | (8854, 8935) | <.0001** |
|  |  |  |  |  |  |
| Fruit | 0 | 10717 (13.8%) | 8348 | (7766, 8930) | Ref |
|  | 1-3 | 54903 (70.6%) | 9072 | (8494, 9650) | <.0001** |
|  | 4+ | 12128 (15.6%) | 9780 | (9196, 10365) | <.0001** |
|  |  |  |  |  |  |
| Water | 0-3 | 38290 (49.3%) | 8415 | (7835, 8995) | Ref |
|  | 4--6 | 30953 (39.8%) | 9206 | (8627, 9786) | <.0001** |
|  | 7+ | 8499 (10.9%) | 9842 | (9255, 10430) | <.0001** |
|  |  |  |  |  |  |
| Screen time | 0--2h | 46664 (60.0%) | 9393 | (8803, 9983) | Ref |
|  | 3-6h | 27853 (35.8%) | 8378 | (7787, 8969) | <.0001** |
|  | 7h+ | 3235 (4.2%) | 7151 | (6543, 7760) | <.0001** |
|  |  |  |  |  |  |
| Mindfulness | 0 mins | 60603 (78.0%) | 8776 | (8184, 9367) | Ref |
|  | 1-9 mins | 9496 (12.2%) | 9528 | (8931, 10124) | <.0001** |
|  | 10+ mins | 7630 (9.8%) | 9481 | (8882, 10081) | <.0001** |
|  |  |  |  |  |  |
| Music | 0 mins | 45271 (58.2%) | 8880 | (8290, 9470) | Ref |
|  | 1-30 mins | 17455 (22.5%) | 8964 | (8372, 9556) | 0.06 |
|  | 31+ mins | 15023 (19.3%) | 9185 | (8592, 9778) | <.0001** |
|  |  |  |  |  |  |
| Alcohol | No | 70747 (95.0%) | 9021 | (8434, 9608) | Ref |
|  | Yes | 3714 (5.0%) | 9078 | (8478, 9678) | 0.42 |
|  |  |  |  |  |  |
| Liquor | No | 73789 (94.9%) | 8940 | (8352, 9528) | Ref |
|  | Yes | 3972 (5.1%) | 9467 | (8866, 10069) | <.0001** |
|  |  |  |  |  |  |
| Marijuana | No | 72673 (93.5%) | 8965 | (8377, 9553) | Ref |
|  | Yes | 5086 (6.5%) | 9146 | (8542, 9750) | 0.017* |
|  |  |  |  |  |  |
| Cigarettes | No | 76252 (98.1%) | 8974 | (8386, 9562) |  |
|  | Yes | 1498 (1.9%) | 9042 | (8402, 9683) | 0.61 |
|  |  |  |  |  |  |
| Illicit Drugs | No | 77391 (99.6%) | 8981 | (8393, 9568) |  |
|  | Yes | 350 (0.4%) | 8725 | (7964, 9487) | 0.31 |
|  |  |  |  |  |  |
| Non-prescribed Pills | No | 76881 (98.9%) | 8985 | (8397, 9572) |  |
|  | Yes | 863 (1.1%) | 8589 | (7915, 9262) | 0.02* |
|  |  |  |  |  |  |
| Wellness Behaviors | 0 Wellness | 11198 (14.4%) | 9024 | (8432, 9617) | Ref |
|  | 1 Wellness | 24449 (31.4%) | 8833 | (8244, 9421) | <.0001** |
|  | 2 Wellness | 22750 (29.2%) | 8860 | (8272, 9448) | 0.002** |
|  | 3 Wellness | 13196 (17.0%) | 9140 | (8549, 9730) | 0.07 |
|  | 4+ Wellness | 6264 (8.1%) | 9424 | (8827, 10022) | <.0001** |
|  |  |  |  |  |  |
| Risk Behaviors | 0 Risk | 39697 (51.0%) | 9338 | (8748, 9928) | Ref |
|  | 1 Risk | 31017 (39.8%) | 8508 | (7918, 9099) | <.0001** |
|  | 2+ Risk | 7143 (9.2%) | 8855 | (8257, 9452) | <.0001** |

Abbreviations—CI: Confidence Interval; Ref: Reference group. * p <.05, ** p <.01.

Demographic factors were controlled in the models.

Supplemental Table 2. Previous day healthy and risky behaviors predicting daily steps within the univariable model.

|  |  | | Estimate | 95% CI | *p* |
| --- | --- | --- | --- | --- | --- |
| Previous day Steps | |  | 0.2 | (0.19, 0.21) | <.0001** |
|  | |  |  |  |  |
| Previous Day Mood | | Sad | 9398 | (8906, 9889) | Ref |
|  |  | Ok | 9405 | (8928, 9882) | 0.92 |
|  |  | Happy | 9065 | (8588, 9541) | <.0001** |
|  | |  |  |  |  |
| Previous Day Sleep | | <4h | 9057 | (8536, 9577) | 0.12 |
|  |  | 4-7 h | 9238 | (8762, 9713) | Ref |
|  |  | 8+ h | 9245 | (8769, 9721) | 0.84 |
|  | |  |  |  |  |
| Previous Day Fruit | | 0 | 8982 | (8503, 9460) | Ref |
|  |  | 1-3 | 9263 | (8791, 9735) | <.0001** |
|  |  | 4+ | 9673 | (9191, 10156) | <.0001** |
|  | |  |  |  |  |
| Previous Day Water | | 0-3 | 9092 | (8617, 9566) | Ref |
|  |  | 4-6 | 9260 | (8786, 9734) | 0.0003** |
|  |  | 7+ | 9533 | (9047, 10019) | <.0001** |
|  | |  |  |  |  |
| Previous Day Screen time | | 0-2h | 9316 | (8840, 9792) | Ref |
|  |  | 3-6h | 9112 | (8634, 9591) | <.0001** |
|  |  | 7h+ | 8841 | (8334, 9348) | <.0001** |
|  | |  |  |  |  |
| Previous Day Mindfulness | | 0 mins | 9135 | (8657, 9612) | Ref |
|  |  | 1-9 mins | 9551 | (9066, 10036) | <.0001** |
|  |  | 10+ mins | 9397 | (8906, 9888) | <.0001** |
|  | |  |  |  |  |
| Previous Day Music | | 0 mins | 9312 | (8836, 9788) | Ref |
|  |  | 1-30 mins | 9211 | (8731, 9690) | 0.05 |
|  |  | 31+ mins | 9088 | (8607, 9569) | 0.0002** |
|  | |  |  |  |  |
| Previous Day Alcohol | | No | 9338 | (8859, 9816) | Ref |
|  |  | Yes | 8673 | (8172, 9174) | <.0001** |
|  | |  |  |  |  |
| Previous Day Liquor | | No | 9278 | (8804, 9753) | Ref |
|  |  | Yes | 8554 | (8057, 9052) | <.0001** |
|  | |  |  |  |  |
| Previous Day Marijuana | | No | 9258 | (8782, 9733) | Ref |
|  |  | Yes | 8871 | (8368, 9373) | <.0001** |
|  | |  |  |  |  |
| Previous Day Cigarettes | | No | 9238 | (8764, 9713) | Ref |
|  |  | Yes | 8963 | (8400, 9526) | 0.08 |
|  | |  |  |  |  |
| Previous Day Illicit Drugs | | No | 9241 | (7895, 9411) | Ref |
|  |  | Yes | 8653 | (8766, 9716) | 0.05 |
|  | |  |  |  |  |
| Previous Day Non-prescribed Pills | | No | 9247 | (8772, 9722) | Ref |
|  |  | Yes | 8632 | (8014, 9250) | 0.003** |

Abbreviations—CI: Confidence Interval; Ref: Reference group. * p <.05, ** p <.01.

Demographic factors were controlled in the models.
